# Apoptosis, Autophagy, NETosis, Necroptosis, and Pyroptosis Mediated Programmed Cell Death as Targets for Innovative Therapy in Rheumatoid Arthritis

**DOI:** 10.3389/fimmu.2021.809806

**Published:** 2021-12-24

**Authors:** Jianan Zhao, Ping Jiang, Shicheng Guo, Steven J. Schrodi, Dongyi He

**Affiliations:** ^1^ Guanghua Clinical Medical College, Shanghai University of Traditional Chinese Medicine, Shanghai, China; ^2^ Department of Rheumatology, Shanghai Guanghua Hospital, Shanghai University of Traditional Chinese Medicine, Shanghai, China; ^3^ Department of Medical Genetics, School of Medicine and Public Health, University of Wisconsin-Madison, Madison, WI, United States; ^4^ Arthritis Institute of Integrated Traditional and Western Medicine, Shanghai Chinese Medicine Research Institute, Shanghai, China

**Keywords:** rheumatoid arthritis, programmed cell death, apoptosis, NETosis, necroptosis, pyroptosis, autophagy

## Abstract

Rheumatoid arthritis (RA) is a chronic inflammatory joint disease that can lead to clinical manifestations of systemic diseases. Its leading features include chronic synovial inflammation and degeneration of the bones and joints. In the past decades, multiple susceptibilities for rheumatoid arthritis have been identified along with the development of a remarkable variety of drugs for its treatment; which include analgesics, glucocorticoids, nonsteroidal anti-inflammatory medications (NSAIDs), disease-modifying anti-rheumatic drugs (DMARDs), and biologic response modifiers (bDMARDs). Despite the existence of many clinical treatment options, the prognosis of some patients remains poor due to complex mechanism of the disease. Programmed cell death (PCD) has been extensively studied and ascertained to be one of the essential pathological mechanisms of RA. Its dysregulation in various associated cell types contributes to the development of RA. In this review, we summarize the role of apoptosis, cell death-associated neutrophil extracellular trap formation, necroptosis, pyroptosis, and autophagy in the pathophysiology of RA to provide a theoretical reference and insightful direction to the discovery and development of novel therapeutic targets for RA.

## Introduction

RA is an autoimmune disease that is usually accompanied by swelling, tenderness, and pain in the joints. It gradually leads to the degeneration of the synovium and joints, often causing disability and premature death (1). RA typically affects one percent of the population, with apparently higher incidences among women than in men (2). The contributing factors and pathogenesis of RA are multifaceted. Studies have shown that it is mainly the production of autoantigens, under the influence of genetic factors, smoking, dust (silica, textile dust), and microbial populations, that activates the adaptive and innate immune system responsible for the pathogenesis of RA. A variety of immune cells release several cytokines and mediators to instigate chronic inflammation of the synovial membrane and destruction of bones and joints during RA progression. These effects thus characterize the immune disorder of RA, which involves immune complex-mediated complement activation, immune response to the post-translationally modified protein autoantigen, dysregulation of cell factor network, and activation of bone destruction-related cells (3–5).

At present, targeted immunotherapy with specific mechanisms (biologically targeted agents and various kinase inhibitors) has made significant progress in the clinical treatment of RA, thereby significantly improving the clinical outcomes in patients. These therapies have been demonstrated to target key molecules and cell nodes that are enriched in the complex mechanism of the disease. However, the treatment failure experienced by some patients administered with immuno-targeted therapies suggests that renewed attention should be duly rewarded to other pathways, such as cell death, for their assessment as prospective targets of intervention (6). Cell death plays different roles under diverse physiological and pathological conditions. Stimulation of death of specific cell types plays specific role depending upon the environmental context. The consequences of cell death in diseases may serve destructive or protective functions, such as those accomplished by the release of inflammatory mediators or protection of host from “harmful or rogue cells”, respectively. For instance, induction of activation-induced cell death (AICD), by the CD44 mediated FasL expression on the surface of T cells through the activation of a tyrosine kinase, IP3 receptor-dependent Ca2+ mobilization, and actin cytoskeletal rearrangements; eliminates self-reactive T cells thereby, suppressing RA (7).

Multiple studies have demonstrated the connection between PCD and RA, especially in the context of various cell populations, including fibroblast-like synoviocytes (FLS), T cells, B cells, monocyte-macrophages, neutrophils, osteoblasts/chondrocytes, and osteoclasts. During the progression of RA, the antagonism of FLS cells to cell death results in excessive proliferation of synoviocytes triggering synovitis. Meanwhile, the imbalance in the cell death of autoimmune T and B cells increases autoimmune and inflammatory responses, whereas the imbalanced cell death of monocyte-macrophages and neutrophils contributes to a variety of pathological reactions associated with RA. On the other hand, enhanced cell death of osteoblasts/chondrocytes and osteoclasts is involved in the RA-associated bone destruction. Thus, the unbalanced cell death of multiple cell types, works in tandem to form a vicious circle that aggravates RA. Accordingly, this review aims to describe the various forms of PCD that may play critical role in the progression of RA, thereby providing insightful reference and direction for further analysis of disease mechanisms and targeted development of innovative therapies for clinical use.

## Apoptosis in the Pathophysiology of Rheumatoid Arthritis

Apoptosis is a form of PCD that has been extensively studied and deliberated (8). Briefly, it essentially mediates cell death *via* two mechanisms involving the exogenous death receptor and the endogenous mitochondrial pathways. In the exogenous death receptor pathway, FasL binds to Fas, resulting in the recruitment of various proteins, and activation of downstream caspase-8, caspase-7, and caspase-3. Caspase-7 activates caspase-6 and the BH3 interacting-domain death agonist (BID) to crosstalk with the endogenous mitochondrial pathway. This results in the activation of a variety of mitochondrial pro-apoptotic proteins that causes accumulation of the B cell lymphoma/leukemia-2 (Bcl-2)-associated X protein (Bax), and Bcl-2 homologous antagonist/killer protein (Bak). Concomitantly, it also leads to the inhibition of anti-apoptotic proteins such as Bcl-2, which cause mitochondrial dysfunction and release of cytochrome c (Cyt c) to activate downstream caspase-9 and caspase-3 that mediate cell death. In addition, the endoplasmic reticulum and lysosomes mediate apoptosis through diverse mechanisms. There are many types of cells that are affected in the course of RA development. One of the important mechanisms contributing to the pathogenesis of RA involves the unbalanced regulation of apoptosis that leads to abnormal expansion or excessive apoptosis of specific cell types. Consequently, it is vital to clarify the relationship between apoptosis and RA especially in the context of various cell types involved.

Although synovial cells are capable to induce apoptosis and reduce cell proliferation through the Fas/FasL pathway, it is quite evident that this mechanism of action is defective during RA. Anti-Fas monoclonal antibody has been proved successful in inducing apoptosis of synovial cells *in vitro*. However, the pro-inflammatory factors such as tumor necrosis factor (TNF)-α and interleukin (IL)-1β have been shown to inhibit Anti-Fas monoclonal antibody-induced apoptosis, potentially by upregulating the Bcl-2-mediated survival, and downregulating the expression of CPP32 and ICH-1L (9). Studies have shown that activators of transcription 3 (STAT3) is an important transcription factor that plays critical role in the regulation of cell survival and apoptosis of various cell types involved in RA. For instance, overexpression of STAT3-YF, a dominant-negative mutant of STAT3, has been shown to inhibit the activation and target gene expression of endogenous STAT3, thereby promoting synovial cell apoptosis (10). Furthermore, heat-shock-protein-70 (HSP70) has been shown to inhibit the apoptotic signal of RA peripheral blood lymphocytes (PBL), resulting in incomplete activation of the CD95/Fas pathway. This phenomenon is characterized by caspase-3/7 activation but no DNA fragmentation, and may thus serve as an important mechanism in the progression of RA (11). Peptidylarginine deiminase IV (PADI4) is responsible for the conversion of peptidylarginine to citrulline. Interestingly, a specific PADI4 SNP haplotype has been reported to strongly induce apoptosis *via* multiple mitochondrial pathways through the downregulation of BCL-XL, upregulation of Bax, and the release of Cyt c into the cytoplasm; thereby providing a possible mechanism underlying the increase of SNP PADI4 activity and highlighting its critical role in the pathogenesis of RA (12). Inconsistently, studies have shown that although excessive TNF-α can attenuate Fas-mediated apoptosis of synovial cells, the effect may not be related to the expression of Fas and Bcl-2. Nevertheless, the benefits of anti-TNF-α therapy in the management of RA are undeniable (13).

**Figure 1:**
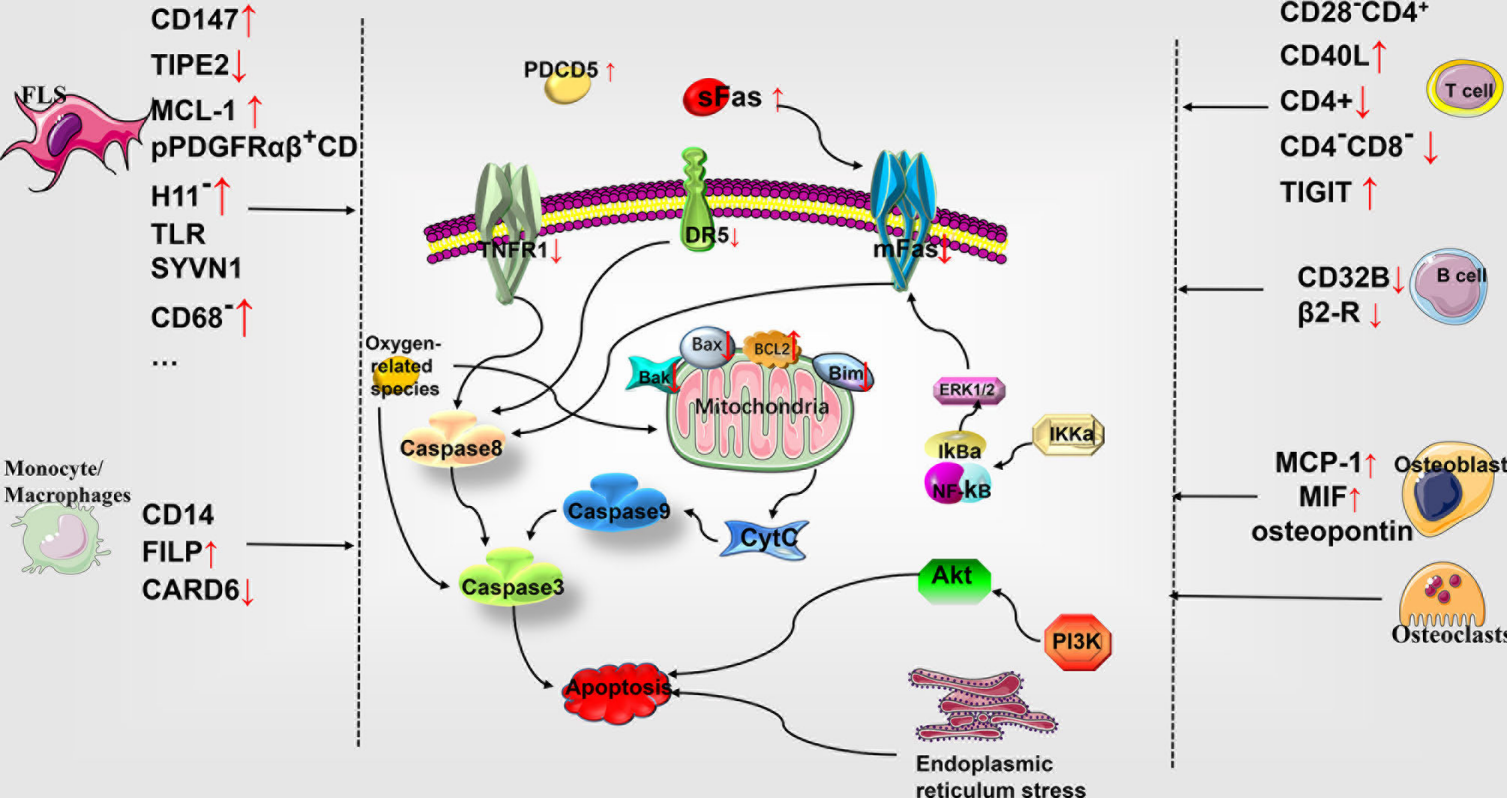
Role of apoptosis in the pathophysiology of rheumatoid arthritis. Multiple cellular subtypes express different molecules in RA, including FLS, monocyte-macrophages, T cells, B cells, osteoblasts, and chondrocytes. The differential gene expression influences the apoptosis of various cells through the molecules related to the exogenous death pathway and the endogenous mitochondrial pathway, thereby affecting the development of RA. MCL-1, myelogenous cell leukemia-1; SYVN1, the E3 ubiquitin ligase synoviolin; CARD6, caspase recruitment domain protein 6; PDCD5, programmed cell death 5; DR5, tumor necrosis factor (TNF)-related apoptosis-inducing ligand (TRAIL) receptor; mFas, membrane-bound Fas; Bcl-2, B cell lymphoma/leukemia -2; Bax, accumulation of Bcl-2–associated X protein; Bak, Bcl-2 homologous antagonist/killer; Bim, Bcl-2 interacting mediator of cell death; Cyt c, cytochrome c; ERK, extracellular signal-regulated kinase; IκBα, intracellular calcium, phosphorylated I-kappa-Bα; TNFR1, tumor necrosis factor receptor 1; PI3K, phosphatidylinositol 3-kinase; TLR, Toll-like receptor; FLS, fibroblast-like synoviocytes.

At present, the commonly used drugs in clinical settings for the treatment of RA include methotrexate, nonsteroidal anti-inflammatory drugs (NSAIDs), and a variety of biological agents, all of which have been shown to be partially involved in the regulation of apoptosis. Methotrexate can increase the local concentration of extracellular adenosine, which can serve as a substrate for the elevated concentration of adenosine deaminase in the synovial fluid of RA patients. This elevated concentration of adenosine may therefore, serve to be beneficial for the treatment of RA by a mechanism that potentially involves the activation of caspase pathway to induce DNA fragmentation and promote FLS cell apoptosis (14). A variety of NSAIDs, such as indomethacin, diclofenac, oxaprozin, and zaltoprofen, can inhibit the proliferation of synovial cells and induce apoptosis. The mechanism underlying this effect may be related to the activation of peroxisome proliferator-activated receptor gamma (PPAR gamma) (15). As far as biological agents are concerned, infliximab is an anti-TNF-α monoclonal antibody that partially promotes caspase-dependent apoptosis and reduces the production of IL-10 and IL-12 (16). Additionally, rituximab reportedly binds to CD20 and induces B cell apoptosis through antibody- and complement- mediated cytotoxicity, thereby treating RA (17). Furthermore, peficitinib, a JAK inhibitor, has been documented to inhibit the autocrine phosphorylation of STAT3 and anti-apoptotic genes in a concentration-dependent manner to promote FLS cell apoptosis (18) (See **Figure 1**).

### Insensitivity to Apoptosis Leads to Abnormal Proliferation of FLS in RA

During the progression of RA, FLS exhibit resistance to apoptosis resulting in synovial hyperplasia that facilitates the release of related cytokines *via* multiple contributing mechanisms. CD147 is highly expressed in FLS during RA, and its extracellular site resists the TNF-α-induced apoptosis by promoting intracellular Ca^2+^ levels through the activation of nuclear factor kappa-light-chain enhancer of activated B cells (NF-κB) signaling pathway (19). TNF-alpha-induced protein-8-like-2 (TIPE2) has been reported to be expressed in very low levels in adjuvant arthritis (AA) model. Moreover, it has been shown to increase the expression of TNF-related apoptosis-inducing ligand (TRAIL) receptor-2 (DR5), promote the activation of caspases, and inhibit the NF-κB signaling pathway to promote FLS cell apoptosis (20). Myelogenous cell leukemia-1 (MCL-1) is highly expressed in the synovial lining and sub-lining layers of FLS during RA. Its expression has been shown to increase in response to IL-1β stimulation and is related to the levels of TNF-α as well as the degree of inflammation. Furthermore, it reportedly inhibits FLS apoptosis by regulating the expression of mitochondrial proteins, such as Bax, Bak, and Bcl-2 interacting mediator of cell death (Bim) (21). Endoplasmic reticulum stress has been documented to exert a pro-inflammatory effect in RA. Studies have shown that FLS antagonizes endoplasmic reticulum stress-induced apoptosis during RA. Endoplasmic reticulum stress is accompanied by the activation of multiple TLR ligands and TLRs, which promotes the production of various cytokines and chemokines (22). Additionally, the E3 ubiquitin ligase synoviolin (SYVN1) has been reported to interact with inositol-requiring enzyme 1 (IRE1) to promote the ubiquitination and degradation of IRE1 and to inhibit FLS cell death in mice due to collagen-induced arthritis (CIA) triggered by endoplasmic reticulum stress. Remarkably, inhibition of SYVN1 reverses this effect (23).

Fas antigen expression is typically detectable in the initial stage of apoptosis, and the elevated expression of Bcl-2 is related to a decrease in apoptosis (24). In RA, the FLS subsets of pPDGFRαβ^+^CDH11^-^ cells are present at a higher proportion in the sub-synovial layer and exhibit increased expression of Bcl-2, decreased expression of TNFR1, resistance to apoptosis, and abnormal proliferation (25). Studies have illuminated some of the ultrastructural characteristics of the apoptotic process in RA associated FLS and determined the modifications in related molecules. Evidently, the apoptotic FLS is present only in the deeper layer of the synovium, while FLS in the remaining layers neither undergo apoptosis nor express Fas antigen (24). Bcl-2 is highly expressed synovial tissues, especially in CD68^-^FLS cells during RA. Inhibition of Bcl-2 leads to changes in mitochondrial permeability, and Cyt c in addition to activation of caspase-9 and caspase-3, DNA fragmentation, and other apoptotic features (26). Soluble Fas (sFas) levels in RA joints have been reported to be elevated and inhibit membrane-bound Fas (mFas) by activating the extracellular signal-regulated kinase (ERK) 1/2, phosphatidylinositol 3-kinase (PI3K), caspase-8, and c-Jun N-terminal kinase (JNK) signaling pathways to enhance the anti-apoptotic effect of FLS. Moreover, it can also promote the cell migration of T cells *via* activation of PI3K. Remarkably, the injection of agonistic anti-Fas antibody has also been documented to evidently improve arthritis in mice with experimentally induced arthritis (27–29). RA patients exhibit higher levels of soluble FasL in the synovial fluid, which is negatively correlated with the levels of vascular endothelial growth factor 165 (VEGF165). This results in the induction of apoptosis in FLS while the reduction in the level of VEGF165 inhibits the migration and chemotaxis of endothelial cells (30). The activation of the ERK and AKT pathways apparently assist FLS in counteracting apoptosis during RA. Estrogen 17β-estradiol has been reported to activate the ERK1/2 signaling pathway to exacerbate RA, promote the TNF-α induced surge in matrix metalloproteinase (MMP)-3, and inhibit the TNF-α induced apoptosis of FLS (31). Imperatorin (IPT) has been demonstrated to reduce the survival of FLS influenced by IL-1β; promote the apoptosis of FLS by increasing the release of mitochondrial cytochrome c, the ratio of Bax/Bcl-2, and other mitochondrial pathways in RA; and alleviates collagen-induced arthritis by reducing the expression of MMP1/3 (32). Similarly, tissue inhibitor of metalloproteinases-3 (TIMP-3) has been shown to inhibit matrix MMP and promote apoptosis. Furthermore, RA- associated FLS ectopically expressing TIMP3 gene exhibit a higher sensitivity to apoptosis by inhibiting the upregulation of Fas/CD95 and activation of NF-κB stimulated by TNF-α (33). The expression of cancerous inhibitor of protein phosphatase 2A (CIP2A) has been reported to activate Akt and inhibit caspase-3, caspase-9, and PARP for promoting the anti-apoptotic effect of RA associated FLS (34). Ceramide, a lipid medium, has been reported to inhibit the phosphorylation and activation of anti-apoptotic protein kinases Akt, MEK, and ERK1/2 in synovial cells stimulated by platelet-derived growth factor (PDGF) (35). TNF-α in RA patients has been documented to stimulate the hypoxia-inducible factor-1α (HIF-1α) activation in FLS through the ERK pathway in addition to transcriptional activation of B-cell activating factor (BAFF), which promotes the survival and inhibits the apoptosis of FLS (36). Lowered pH has been shown to activate the acid-sensing ion channel 3 (ASIC3) expressed in FLS, resulting in an increase in Ca^2+^ and a decrease in p-ERK levels, thereby inducing cell death through calcium-dependent pathways (37). Similarly, a pH 5.5 solution and capsaicin, a transient receptor potential vanilloid 1 (TRPV1) agonist, have been reported to increase the expression of *TRPV1* mRNA, accompanied by increased Ca^2+^, reactive oxygen species (ROS), and depolarization of the mitochondrial membrane potential to promote synovial cell apoptosis (38), However, a pH 6.8 solution triggered activation of phospholipase C, intracellular release of Ca^2+^, nuclear translocation of NF-κB, and inhibition of ROS production, results in the mitigation of capsaicin-induced apoptosis of synovial cells (39). Thus, the decrease in the pH may prove to be beneficial in limiting the proliferation of the synovium and subsequent joint damage associated with RA.

There are many oxygen-related species in RA, including nitric oxide (NO), ROS, and their products, which affect the apoptosis of FLS through different mechanisms. NO is elevated in RA synovial fluid, and serum directly inhibits caspase-3 activation and defends the proliferation of synovial cells (40). In addition, studies have shown that 3-nitrotyrosine, a marker of reactive nitrogen species, induces chondrocyte mitochondrial dysfunction and caspase-independent apoptosis through a calcium-dependent mechanism (41). Menthol, a specific agonist of transient receptor potential melastatin subtype 8 (TRPM8), promotes Ca^2+^ influx and TRPM8 activation to generate ROS, which induces synovial cell apoptosis (42). In addition, a prolonged O_2_/Ca^2+^-supporting phototherapy hydrogel (POGel) system has been shown to enhance the direct killing effect of singlet oxygen on cells and promote articular cartilage regeneration. The resultant calcium peroxide (CaO_2_) induces mitochondrial Ca^2+^ overload and endoplasmic reticulum Ca^2+^ disorder, triggering Ca^2+^-related FLS apoptosis and immunogenic cell death to alleviate RA-related synovial proliferation and joint destruction (43). Mitomycin C has been demonstrated to inhibit FLS proliferation and promote FLS apoptosis by increasing ROS production, inducing mitochondrial dysfunction, and increasing the release of mitochondrial cytochrome c, the ratio of Bax/Bcl-2, caspase-9, caspase-3, and cleavage of poly(ADP-ribose) polymerase (44). Moreover, apigenin, a dietary plant-flavonoid, has been reported to induce FLS apoptosis and activate ERK1-2/caspase-3/caspase-7 signaling by generating excessive ROS (45). Similarly, hempseed oil has been shown to promote apoptosis of MH7A cells in a time- and dose- dependent manner, accompanied by lipid accumulation, increased ROS production, and elevated expression of an endoplasmic reticulum stress protein, the EBP-homologous protein (CHOP) (46). Furthermore, resveratrol, a phytoalexin, has been reported to induce MH7A cell apoptosis by stimulating the release of Cyt c, activating caspase-3 and caspase-9, up-regulating the transcription of the NAD-dependent deacetylase- *sirtuin 1*, and downregulating the transcription of survival factor- *Bcl-xl* (47). Interestingly, geldanamycin (GA) can promote ROS production in RA associated FLS and induce apoptosis *in vitro* (48). In addition, programmed cell death 5 (PDCD5) and P53 may also serve as critical apoptotic targets. In RA patients, PDCD5 levels in the serum and synovial fluid are elevated, which may be attributed to the negative association of reduced sensitivity of FLS to apoptosis and its interaction with CRP, ESR, IL-17, and TNF-α (49, 50). The expression and nuclear translocation of PDCD5 have been reported elevated in the synovial tissue and FLS in RA. Overexpression of PDCD5 causes triptolide to promote caspase-3 expression and apoptosis in FLS (51). In addition, daphnetin has been shown to reduce the expression of DNMT1, DNMT3a, and DNMT3b and mediate the demethylation of pro-apoptotic genes- *DR3*, *PDCD5*, *FasL*, and *P53*, resulting in augmented apoptosis of synovial cells in CIA (52). The p53 upregulated modulator of apoptosis (PUMA) has been documented to promote apoptosis of FLS cells in a potentially P53 independent manner. Thus, gene therapy targeting PUMA can also serve as a promising treatment strategy for RA (53). However, studies have shown that the abnormal function of P53 also contributes to the abnormal proliferation of FLS (54), and calpain inhibitor 1 (ALLN) can induce P53 protein expression in synovial cells and cooperate with hydrogen peroxide to promote the apoptosis of synoviocytes (55). Furthermore, TNF-α depletes CREB binding protein (CBP)-induced P53 acetylation state, thereby attenuating its transcriptional activity. Consequently, the overexpression of CBP assists in activating and restoring the transcriptional activity of P53, which ultimately increases the TNF-α-induced apoptosis of synoviocytes in RA (56).

A synthetic second mitochondria-derived activator of caspase (Smac 066) has been reported to inhibit the cell survival promoting inhibitors of apoptosis proteins (IAPs), thereby demonstrating a robust pro-apoptotic effect on FLS in RA. Its mechanism may involve the activation of caspase-3 and caspase-8, the downregulation of IAPs, and the upregulation of IGFBP-5 (57). Jüngel et al. have shown that trichostatin increases the *in vitro* sensitivity of FLS to TRAIL-induced apoptosis by upregulating p21Waf1/Cip1 (58). Thus, targeting the differentially expressed apoptosis promoting molecules and promoting the generation of oxygen-related species may serve as a potential measure to reduce the proliferation of RA synovium and increase the sensitivity of FLS to apoptosis. However, it is worth noting that FLS with a high proliferation state is significantly less sensitive to Fas-induced apoptosis than FLS with a low proliferation rate. Additionally, the sensitivity of FLS in the S or G2/M phase to TRAIL-induced apoptosis has been reported to be substantially lower than that in the G0/G1 phase (59).

### Abnormal Expansion of Autoimmune T Cells Mediates Inflammation of the Synovium

In RA, elaborate studies have shown that a large number of T cells infiltrate the synovium; interact with other cells, including dendritic cells (DC), monocytes, macrophages, and FLS; release a large number of inflammatory and a small number of anti-inflammatory mediators; and jointly mediate the chronic inflammation of the synovium. Evidently, this RA associated abnormal expansion of autoimmune T cells may be caused by increased homing as well as retention, and/or an imbalanced regulation of apoptotic pathway in these cells (60). Among the CD4^+^ T lymphocytes in RA patients, CD28^-^CD4^+^ T cells highly express Bcl-2, which leads to atypical clonal expansion of autoimmune T cells [60]. In addition, the circulating T cell populations of RA patients highly express CD40L and interact with CD40 to activate B cells and decrease CD32B expression, which inhibits the apoptosis of autoimmune memory B cells. Furthermore, this effect can be enhanced by IL-4, IL-10, and IL-21 (61). The RA auto-antibody B7-H1 exerts a dual regulatory effect on CD4^+^ T cells in promoting proliferation as well as apoptosis. It has been reported to stimulate the proliferation of T cells and the release of IL-10, accompanied by the upregulation of TNF apoptosis-inducing ligand and caspase-3, resulting in abnormal responses of T cells associated with aggravated RA (62). Treg cells can regulate a variety of immune cells to prevent autoimmune destruction by various mechanisms. Rapetti et al. have shown that failure to inhibit CD4^+^ T cell activation is concomitant with the failure of Treg cells in inhibiting the B cell activation and production of pro-inflammatory factors and autoantibodies in RA. Thus, the reduced ability of Treg cells in regulating the apoptosis of B cells decreases the expression of Fas in B cells, rendering them resistant to apoptosis (63).

Several studies have used experimentally induced arthritis animal models to study the relationship between T cells and apoptosis and delineate the possible underlying mechanisms. Studies have shown that activation of DCs by the receptor activator of nuclear factor (NF)-κB ligand (RANKL) is accompanied by a decrease in CD4^+^ T, B, and CD4^-^CD8^-^ double negative T cells in MRL/LPR experimental autoimmune mice. Further, the TNF-related apoptosis-inducing ligand-receptor 2 (TRAIL-R2) of T cells has been shown to directly interact with TRAIL on DC cells to induce Fas-independent apoptosis as a new mechanism for maintaining peripheral immune tolerance (64). Overexpression of the *Galectin-1* and downregulation of *Galectin-3* has been reported to inhibit arthritis in the CIA mice and significantly reduce multiple disease activity scores, T cell infiltration, and microvessel density by inducing lymphatic T cell apoptosis (65). The P2RX7 in the K/BxN autoimmune arthritis model has been reported to antagonize the production of autoimmune B cells and autoantibodies by promoting cell death of T follicular helper cells. In contrast, the activation of TIGIT, a T cell exhaustion marker, has been demonstrated to upregulate the resistance factor of apoptosis and promote T cell survival, thereby counteracting the effect of P2RX7 (66). The arthritis resistance of HLA-DRB1*0402 expressing mice has been documented to be possibly related to the loss of autoimmune T cells, a higher number of regulatory T cells, and increased activation-induced apoptosis in these mice (67). In addition, the CD4^-/-^ transgenic mice with HLA-DQ8 are resistant to CIA, whereas the CD8^-/-^ HLA-DQ8 harboring mice exhibit a higher disease incidence and severity than the control group. These results indicate that CD4 is a necessary factor for initiating CIA in DQ8 transgenic mice. Although CD8^+^ T cells fail to induce CIA in DQ8 transgenic mice, they exert a protective effect against apoptosis (68). Thus, inducing autoimmune T cell apoptosis may serve as a potent strategy to treat RA. Galactoxylomannan (GalXM) acts on CD45RO T cells in RA to inhibit the interaction of CD45 phosphatase with T cells, the production of IL-17A, and the activation of STAT3 and caspase-3 to promote apoptosis of memory T cells (60). Furthermore, lentiviral particles facilitated expression of mFasL has been shown to activate caspase-3 and caspase-9 and release Cyt c by binding to Fas on the surface of T cells to induce apoptosis (69). Moreover, a new type of recombinant fusion protein scFvCD7, developed on the pretext that CD7 targets sFasL that specifically binds to T cells, can selectively activate the Fas/FasL signaling pathways in Th1 and Treg cells to induce autoimmune T cell apoptosis and thus, possesses a promising therapeutic value for RA (70).

### Imbalanced Regulation of Apoptosis in Osteoblasts/Chondrocytes and Osteoclasts Mediates Bone Destruction in RA

Many RA associated mechanisms lead to excessive apoptosis of osteoblasts or chondrocytes, and the antagonism of apoptosis demonstrated by osteoclasts contributes significantly to bone destruction in RA. Therefore, protecting osteoblasts or chondrocytes and promoting osteoclast apoptosis may serve as promising treatment strategies for RA. Incubation of human chondrocytes with the synovial fluid of RA leads to increased secretion of MCP-1 and MIF, which promotes chondrocyte apoptosis (71). The expression of FasL in activated peripheral blood mononuclear cells combined with Fas expressed on the human osteoblast cell line MG63 and primary osteoblasts has been shown to result in osteoblast apoptosis and induction of bone loss in RA (72). Furthermore, the chondrocyte apoptosis in mouse arthritis induced by anti-type II collagen antibodies and lipopolysaccharide (mAbs/LPS) has been shown to be potentially mediated by osteopontin. Additionally, the inhibition of osteopontin results in the downregulation of TNF-α induced up-regulation of caspase-3, while the overexpression of osteopontin reverses this effect (73). Excessive ROS generation in the synovial fluid or plasma of RA patient leads to protein oxidation resulting in the generation of advanced oxidation protein products, which function as pro-inflammatory mediators. These products have been reported to exert pro-apoptotic effects on chondrocytes *in vitro via* the RAGE-nicotinamide adenine dinucleotide phosphate (NADPH) oxidase/poly (ADP-ribose) polymerase-1 (PARP-1)-mediated redox-dependent pathway (74). TNF-α can inhibit the apoptosis-promoting effect of NO on chondrocytes through the NF-κB/COX2 pathway (75). Evidently, YS-51S, a synthetic isoquinoline alkaloid, inhibits the oxidation of peroxynitrite on Cyt c, the expression of inducible nitric oxide synthase (iNOS) and NO-mediated apoptosis of osteoblasts (76). Furthermore, the extracts of *Emblica officinalis* have been shown to induce osteoclast apoptosis and compete with NF-κB binding, resulting in decreased secretion of IL-6 (77). Additionally, fraxetin has been reported to inhibit the TNF-α and IL-1β mediated expression of Fas, upregulate the expression of FLIP, and inhibit caspase-3 and caspase-8 by inhibiting osteoblast apoptosis (78).

### Decreased Apoptosis of Pro-Inflammatory Cell Subtypes Exacerbates the RA Associated Inflammation and Bone Destruction

It is well-established that there is an increase in the pro-inflammatory cell populations during RA. As has been discussed so far, increasing the sensitivity of these “harmful” cell populations to apoptosis can be helpful in alleviating RA. CD14 monocytes in the peripheral blood and synovial fluid of RA patients have been reported to be resistant to spontaneous apoptosis, which may be due to the increased expression of miR-155 resulting in the inhibition of apoptotic factors- caspase-10 and APAF1 (79). Moreover, the upregulation of the survival protein FLIP in macrophages has been shown to protect macrophages by inhibiting Fas-mediated apoptosis, thereby contributing to inflammatory diseases (80). Encouragingly, thalidomide has been shown to induce monocyte apoptosis through endogenous mitochondrial pathways by promoting the release of Cyt c, activating caspase-3, caspase-9, and NF-κB; and inhibiting Akt-1 activity thereby facilitating the treatment of RA (81). In addition to the effects of sex hormones on FLS, androgens have been documented to increase *Bax*, *Fas*, and *PARP* expression in the THP-1 mononuclear macrophage cells. Multiple studies have suggested that sex hormones can regulate apoptosis of macrophage-like cells implicating their potential therapeutic effect on RA (82, 83). Short-term treatment with 100-300 ng/ml Cyclosporin A, an immunosuppressant of RA, has been shown to induce apoptosis of THP-1 monocyte cells (84). Moreover, the LPS-induced expression of caspase recruitment domain protein 6 (CARD6) is found to be suppressed in macrophages which results in the augmented release of pro-inflammatory factors. In contrast, overexpression of CARD6 results in decreased expression of pro-inflammatory factors and chemokines in addition to inhibition of TNFR1/tumor necrosis factor receptor-associated factor-2 (TRAF2)/NF-κB signaling pathway, which alleviates not only the TNF-α induced inflammation and apoptosis of macrophages but also the level of bone destruction in CIA mice. Furthermore, it inhibits the activation of cleaved caspase-3, indicating that CARD6 plays an anti-inflammatory and anti-apoptotic role in RA (85). The high expression of SH3s may be involved in the protection and survival of synovial cells as well as stimulation of cell proliferation (86). Chronic inflammation in RA causes neutrophils to activate and release hydrogen peroxide along with the enzyme myeloperoxidase. These phenomena ultimately results in the formation of hypochlorous acid (HOCl), which is found to be elevated in RA synovial fluid. HOCl has been reported to induce apoptosis of human mesenchymal progenitor cells (MPCs) by increasing Bax-dependent mitochondrial permeability and the AIF-/EndoG- dependent pathway (87). Cell death and cartilage destruction in RA synovium are potential sources of mitochondrial antigen release that induce local antigen-driven IgG2/lambda B cell and inflammatory responses (88). The expression of β2-R in the lymphatic B cells of patients with RA has been documented to be significantly reduced as compared to that in healthy controls. The expression of β2-R has been reported to be negatively correlated with disease progression, which may be related to the lowered induction of cAMP and cell death (89). In addition, studies have shown that the expression of sphingosine 1-phosphate in the lymphoblastic B cell lines of RA patients increases through the G protein-coupled receptor-mediated activation of PI3K signaling, leading to the antagonism of Fas-mediated apoptosis (90).

## Autophagy in the Pathophysiology of Rheumatoid Arthritis

**Figure 2:**
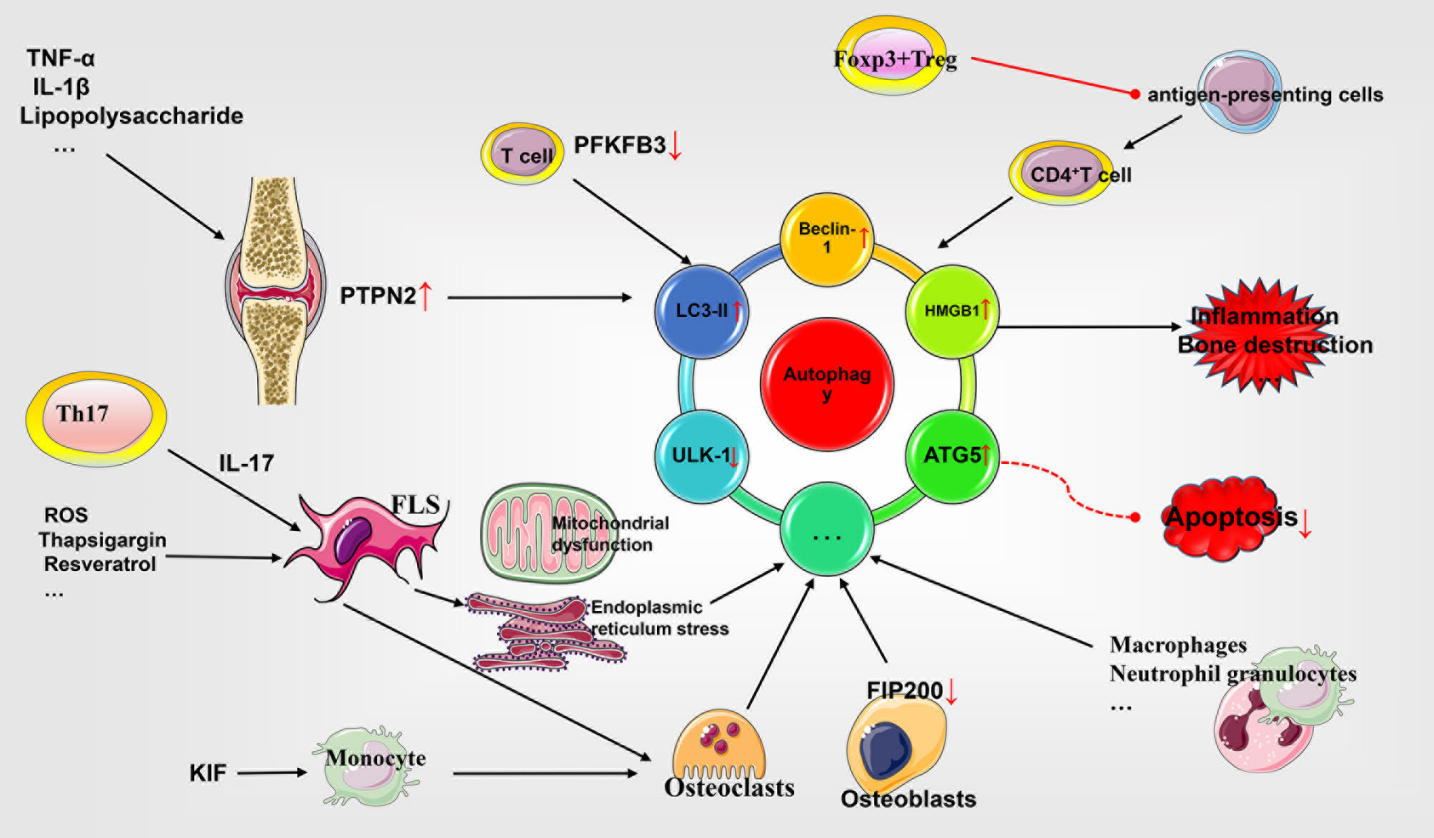
Role of autophagy in the pathophysiology of rheumatoid arthritis. Autophagy and apoptosis appear to be antagonistic to each other. FLS enhances autophagy by expressing different molecular patterns and antagonizes apoptosis to promote synovial cell proliferation. In addition, insufficient autophagy flux in T cells causes premature senescence and apoptosis. Autophagy of antigen-presenting cells is involved in inflammation. Autophagy of osteoblasts and osteoclasts affects the process of bone destruction. Other cells affect RA through different mechanisms. TNF, tumor necrosis factor; IL, interleukin; PTPN2, phosphatase nonreceptor type 2; ROS, reactive oxygen species; LC3, light chain 3; ULK-1, UNC51-like kinase 1; FLS, fibroblast-like synoviocytes; PFKFB3, 6-phosphofructo-2-kinase/fructose-2,6-bisphosphatase 3; FIP200, focal adhesion kinase family interacting protein of 200 Kd; HMGB1, high mobility group box 1; ATG5, autophagy related 5.

Autophagy is a self-eating phenomenon responsible for the elimination of impaired organelles, abnormal and old nonfunctional proteins, and other intracellular substances *via* lysosomal degradation; for their recycle and efficient use in important cellular functions, especially in response to stressful physiological conditions (91). The detailed molecular mechanisms and characteristics of autophagy have been widely reviewed, and mainly involve the mTOR- mediated formation of autophagosomes and autophagolysosomes and the reutilization of resultant products (92, 93). The expression of autophagy associated proteins in the synovial tissues of RA patients has been reported to be remarkably increased (Beclin1, ATG5, LC3) and significantly correlated with the serum levels of inflammatory markers (CRP, ESR) and autoantibodies (cyclic citrullinated peptide, CCP; and rheumatoid factor, RF). Moreover, the anti-TNF-α therapy and IL-6R inhibitor treatment have been documented to result in decreased levels of autophagy (94, 95). Hydroxychloroquine and chloroquine, the commonly used therapeutic drugs for RA, have also been demonstrated to inhibit autophagy by preventing immune activation and cytokine production of various cells and regulating the expression of CD154 on T cells (96). Thus, the phenomenon of autophagy is closely related to the progression of RA. Accordingly we have primarily discussed the relationship between autophagy and various cell subtypes, including FLS, T cells, antigen-presenting cells, osteoblasts, osteoclasts, macrophages, and neutrophils (See **Figure 2**).

### Autophagy Inhibits the FLS Apoptosis and Enhances Synoviocytes Proliferation in RA

Autophagy in RA has a close association with apoptosis, wherein autophagy exhibits an antagonistic effect on apoptosis. Assessment of synovial tissues from patients with RA and osteoarthritis (OA) has revealed that while the apoptosis of RA synovial tissues was reduced, the expression of autophagy related proteins, Beclin-1 and LC3, were highly augmented. These findings, thus suggest a significant negative correlation between apoptosis and autophagy (97).Furthermore, studies have shown that TNF-α activates the NF-KB signaling in FLS and enhances autophagy, which in turn enhances the resistance of FLS to anti-TNF-α therapy for RA (98, 99). Similarly, FLS induces the formation of autophagosomes and resistance to methotrexate in RA by enhancing the expression of HMGB1 and Beclin-1 (100). Thus, various mechanisms promote the autophagy of FLS and antagonize cell apoptosis while also enhance the proliferation ability of FLS. The expression of protein tyrosine phosphatase non-receptor type 2 (PTPN2) in the synovial tissue of RA patients is higher than that in OA patients. Moreover, TNF-α, IL-1β or lipopolysaccharide have been reported to stimulate the upregulation of PTPN2, which aids in the prevention of apoptosis and increasing the level of autophagy in FLS. However, silencing of PTPN2 aggravates the production of IL-6, the mechanism underlying which requires further experimental elucidation (101). The upregulation of dynamin 1-like protein (DNM1L) has been shown to promote the survival of FLS. It also stimulates inflammation by increasing the expression of autophagy protein LC3B, ROS production, inhibiting cell apoptosis, and regulating AKT/IKK/NFKBIA/NF-Kb signaling (102). Furthermore, the IL-17 secreted by the Th17 cells have been reported to induce mitochondrial dysfunction and autophagy in FLS by activating STAT3, thereby antagonizing the apoptosis of FLS in RA (103, 104). Resveratrol has been demonstrated to reduces autophagy related proteins such as Beclin-1 and LC3A/B, thereby augmenting mitochondrial dysfunction as well as FLS apoptosis, and alleviating the symptoms of adjuvant-arthritis (AA) in rats (105). Knockdown of PADI4, a genetic susceptibility gene for RA, has been shown to inhibit FLS autophagy while promoting apoptosis (106). Exogenous administration of ROS to stimulate RA FLS expressing TLR4 receptors induces a vital state of oxidative stress state and promotes the release of high mobility group box 1(HMGB1) and autophagy, which has an antagonistic effect on apoptosis (107).The endoplasmic reticulum (ER) stress inducer thapsigargin reportedly results in lower expression of the ER stress protein CHOP in FLS and induces autophagy, through the formation of autophagosomes, Beclin-1 expression, and LC3-II transformation, to increase the resistance of FLS to cell death caused by ER stress, such as apoptosis in RA (108). In addition, thapsigargin promotes FLS cell death in a caspase-3 independent manner, accompanied by remarkable aggregation of p62-positive polyubiquitinated protein and reduced expression of autophagy-linked FYVE protein (AFLY); suggesting that autophagy may have a dual role in regulating FLS survival and proliferation in RA (109).

### Autophagy Regulates T Cell Subsets in RA

The increased autophagy of FLS has been shown to antagonize apoptosis, while the decreased autophagy of T cells results in augmented apoptosis, leading to chronic T cell loss and lymphocytopenia, which are risk factors for RA. The expression of 6-phosphofructo-2-kinase/fructose-2, 6-bisphosphatase 3 (PFKFB3) has been found to be reduced in RA associated T cells. Consequently, it is easily reprogrammed by metabolism and lack of energy and ROS and autophagy, leading to senescence and apoptosis. Overexpression of PFKFB3 has been reported to promote glycolytic flux and regulate autophagy, thereby preventing excessive T cell apoptosis (110–112). In addition, autophagy may be attributed to the conference of memory to the inflammatory CD4^+^ T cells, which leads to continuous recognition and activation of autoantigens that promotes inflammation in RA. Furthermore, it has been shown that inhibition of MYC may serve as a putative mechanism for the augmentation of autophagy in RA (113). Simultaneously, the increase in the level of autophagy has been demonstrated to inhibit the apoptosis of inflammatory CD4+ T cells and prolong cell survival time, further facilitating RA progression (114).

### Autophagy Increases Auto-Antigen Presentation by Antigen-Presenting Cells

Various studies support the notion that autophagy is involved in the process of autoantigen presentation by APC cells to CD4^+^ T cells. For instance, starvation of B cells results in the production of citrullinated peptides, which is also accompanied by an increase in the expression of autophagy protein ATG5. Furthermore, the treatment of B cells by autophagy inhibitor abrogates the presentation of citrullinated peptides. Nevertheless, it does not affect other unmodified peptides (115). Another study has shown that macrophages, dendritic cells, and B cells undergo autophagy to produce citrullinated peptides after the antigen contacts the autophagic vesicle (116). Further, it has been reported that induction of FLS autophagy *in vitro* is accompanied by peptidyl arginine deiminase 4 activation and protein citrullination. This was further corroborated by a significant positive correlation between autophagy and anti-CCP levels in RA patients (117). Additionally, Foxp3^+^ Treg cells have been reported to inhibit the autophagy as well as the autoimmune response of dendritic cells in a cytotoxic T-lymphocyte-associated protein 4-dependent (CTLA4) dependent manner. The underlying mechanism for this phenomena possibly involves the interaction between CTLA4 and PI3K/AKT/mTOR axis, which leads to reduced autophagy levels (118). In addition, studies have shown that autophagy may be involved in the carbamylation of FLS proteins, a non-enzymatic post-translational modification mechanism, which results in their accumulation and subsequent participation in the pathogenesis of RA. However, the specific mechanism underlying this phenomenon is not yet clear (119).

### Regulation of Autophagy in Osteoblasts and Osteoclasts in RA

The autophagy defect of osteoblasts leads to suppression of bone formation. It has been reported that defective focal adhesion kinase family interacting protein of 200 kDa (FIP200) in osteoblasts results in insufficient autophagy, accompanied by abnormal expression of p62, defects in autophagy flux, and ultimately lower bone mass (120). Similarly, defects in ATG7, the key autophagy protein in osteoblasts, have been documented to decrease osteoblast formation, matrix mineralization, and secretion of osteoprotegerin TNFRSF11B/OPG; and augment osteoclast population, secretion of TNFSF11/RANKL, and endoplasmic reticulum stress, to promote apoptosis (121). The increased autophagy in osteoclasts leads to enhanced production, which mediates bone destruction. Remarkably, the defects of autophagy-related proteins have been shown to facilitate prevention of bones and joints destruction. For instance, TNF-α has been reported to stimulate the expression of osteoclast autophagy proteins Beclin1, and ATG7; activate autophagy; and regulate osteoclast differentiation, and bone resorption in RA (122, 123). The expression of the autophagy receptor optineurin in FLS has been documented to be decreased under the action of a variety of inflammatory factors (e.g., TNF-α). Subsequently, it reportedly stimulates the expression of RANKL and the differentiation of osteoclasts (124). Kruppel-like factor 2 (KIF) negatively regulates the expression of the autophagy protein Beclin1 in monocytes and induces osteoclast differentiation and autophagy levels to enhance joint inflammation and bone destruction (125). Deficiency of the autophagy protein Afly leads to the up-regulation of TRAF6 in osteoclasts and regulates RANKL-induced osteoclastogenesis (126).

### Autophagy Regulates Other Cells Associated With RA and Targeting Autophagy Significantly Alleviates RA

RA associated macrophages highly express SNAPIN, mainly to maintain autophagy and healthy lysosome function and prevent proton leakage from the lysosomes (127). A variety of cell surface proteins such as CD244 bind to autophagy proteins such as Vps34 and Beclin-1, to facilitate inhibition of autophagy. The down-regulation of CD244 has been shown to augment autophagy, which is associated with the severity and prognosis of RA (128). The concentrations of IL-6, IL-8, IL-10, and MCP-1 in RA synovial fluid are reportedly elevated and mediate the autophagy of neutrophils through their corresponding cytokine receptors (129). The autophagy level in the peripheral blood mononuclear cells has been reported to be elevated in RA with concomitant decrease in apoptosis. Moreover, anti-TNF-α therapy has been documented to decrease the levels of autophagy protein LC3-II, which also correlates with the DAS28 score, thereby resulting in augmented apoptosis (130).

Many studies have shown that targeting autophagy can significantly improve the prognosis of RA through a variety of mechanisms. For instance, celastrol has been shown to inhibit sarcoplasmic/endoplasmic reticulum calcium ATPase pump (SERCA) induced autophagy by regulating the Ca^2+^/calmodulin-dependent kinase kinase-β (CaMKKβ) -AMP-activated protein kinase (AMPK) -mTOR pathway. Moreover, such induction of autophagy is accompanied by the alleviation of arthritis symptoms in rats with adjuvant-induced arthritis (AIA) (131). The autophagy-associated protein, microtubule-associated protein light chain 3b (LC3b) has been reported to be upregulated, and while UNC51-like kinase 1 (ULK-1) is downregulated in experimental arthritis mice. In contrast, the extract of tomorou, an indigenous herb of Hunza-Nagar Valley, Pakistan has been reported to inhibit LC3B protein by up-regulating caspase-3. Furthermore, its water and ethyl acetate extracts also result in the normalization of abnormal ULK-1 protein levels, thereby alleviating arthritis symptoms (132).

It is worth noting that targeting AKT-related pathways to regulate autophagy is also a potential direction for the treatment of RA. For instance, dexamethasone has been reported to induce autophagy by increasing the ROS levels in human chondrocytes by regulating the AKT/FOXO3 signal pathway to inhibit apoptosis (133). Furthermore, tangeretin and 5-hydroxy-6,7,8,3’,4’-pentamethoxyflavone have also been shown to inhibit autophagy by regulating ROS levels in synovial cells and AKT/mTOR signaling, thereby inhibiting the pathological changes associated with bovine type II collagen-induced arthritis in animals (134). Moreover, astragalus polysaccharides have been shown to regulate FLS autophagy and enhance the expression of pro-apoptotic proteins such as Bax and Caspase-3, through PI3K/AKT/mTOR signalling to promote apoptosis of FLS and improve RA prognosis (135). Remarkably, the combination therapy of pterostilbene and physical exercise has been documented to inhibit the proliferation and IL-1 stimulated apoptosis of rat synovial cells through the PI3K/AKT/NF-KB signaling pathway and enhance the level of autophagy to alleviate the symptoms of experimental arthritis in rats (136). Similarly, autophagy inhibitors have also been demonstrated to directly reduce the inflammatory response of experimental arthritis rats, inhibit the proliferation of FLS through the PI3K/AKT pathway, and promote their apoptosis (137).

In subsequent sections, we predominantly discuss three types of PCD: necroptosis, pyroptosis and NETosis. Notably, the research related to these three types of PCD in the context of RA is not as advanced as in case of apoptosis and autophagy as discussed in previous sections. Hence, further sophisticated clinical pre-experiments and clinical trials are essential to understand the in-depth role of PCD in RA prognosis.

## NETosis as a Source of Citrullinated Autoantigens and an Inflammatory Driver in Rheumatoid Arthritis

Neutrophils are white blood cells that mature in the bone marrow and are released into the circulation. They are characterized by a granular cytoplasm and lobulated nuclei. Neutrophils in healthy humans have a short lifespan. Instigation with a variety of anti-inflammatory or pro-inflammatory signals triggers the chemotaxis and accumulation of neutrophils at the inflammation site under the influence of a variety of cytokines. This ultimately results in the occurrence of apoptosis that participates in the process of inflammation subsidence (138, 139). An important physiological defense mechanism of neutrophils is the production of neutrophil extracellular traps (NETs), which are extracellular network-like structures that contain DNA, histones, neutrophil proteins (alarmins), myeloperoxidase (MPO), neutrophil elastase (NE), and cathepsin G and are designed to eliminate harmful pathogens (138, 140, 141). For instance, neutrophils themselves participate in the enhanced citrullination of histones by expressing the peptidylarginine deiminase (PAD)2/PAD4 enzymes, whereas NADPH oxidase (NOX) is involved in the generation of ROS, and a variety of serines contribute to chromatin remodeling. The process also involves the participation of proteases, including neutrophil elastase and cathepsin G, thereby resulting in NETosis which releases an array of inflammatory mediators to drive RA progression (142–144).

**Figure 3:**
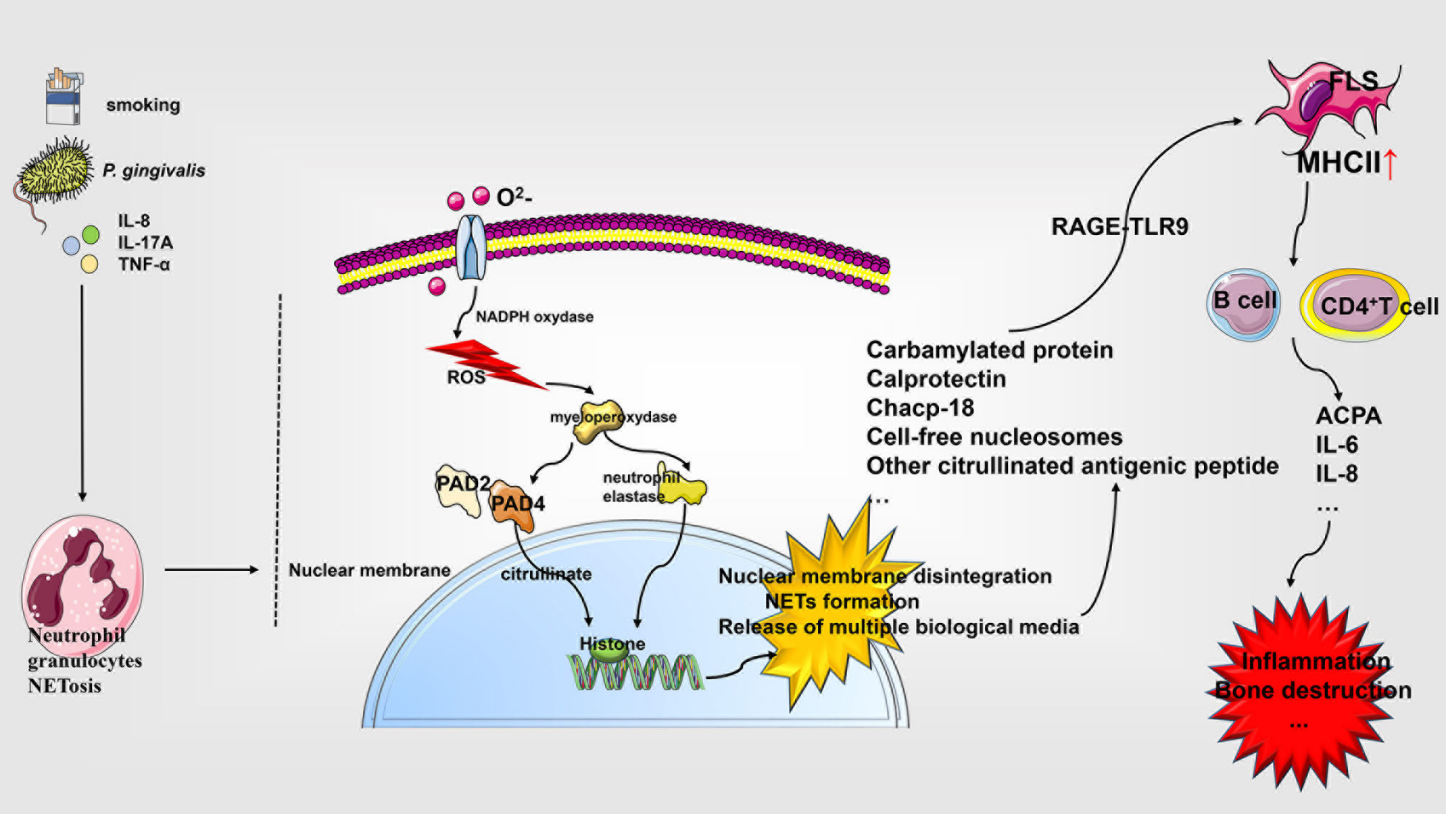
Role of NETosis in the pathophysiology of rheumatoid arthritis. Neutrophils respond to diverse stimuli for developing NETosis, which starts with the NADPH oxidase complex and promotes ROS production. ROS activate the hydrolytic activity of PAD4 and neutrophil elastase in an MPO-dependent manner, cleaving the histones over-citrullinated by PAD4. This triggers a series of events, including the rupture of the nuclear membrane, the formation of NETs, and the release of various biological agents, which interact with a variety of cells affecting RA progression. IL, interleukin; PAD, peptidylarginine deiminase; chcap-18, cathelicidin human cationic antimicrobial protein-18; NETs, neutrophil extracellular traps; NADPH, nicotinamide adenine dinucleotide phosphate; TLR, Toll-like receptor; MHCII, MHC class II; ACPA, anti-citrullinated protein antibodies; FLS, fibroblast-like synoviocytes; ROS, reactive oxygen species.

The susceptibility factors in patients with RA include microorganisms associated with periodontitis and smoking (145). Among them, *P. gingivalis* has been reported to express PAD enzyme homologues to induce the production of citrullinated antigens in patients with periodontitis, which enhance NETosis, further promoting RA. A remarkable reduction in the serum levels of carbamylated protein (CarP) and NETs post periodontal treatment, further substantiates the possible association between RA and periodontitis (146–149). In addition, studies have shown that in the lungs of smokers, PAD2 and PAD4 expressed by neutrophils can citrullinate the cathelicidin human cationic antimicrobial protein-18 (chcap-18) that can be potentially externalized by NETosis to promote RA (146, 148, 150, 151). Some studies have identified that the production of some extracellular citrullinated antigens in RA may be associated with the inflammatory process of NETosis in neutrophils. The active PAD2/4 enzyme is evidently released into the synovial fluid of RA patients to promote the generation of autoantibodies, which results in inflammation. However, the phenomenon has no association with the apoptosis process of neutrophils (152, 153). Activated neutrophils are significantly elevated in synovial fluid of RA patients. Studies suggest that they might be involved in responding to the synergistic effect of the immune complex and complement system; resulting in excessive NETosis formation and release of a variety of inflammatory mediators to mediate inflammation and joint destruction (148, 154, 155). The spontaneous increase of neutrophil population in RA patients is associated with increased ROS production, MPO expression, and citrullination mediated by PAD4 activity. The NETosis derivatives including plasma cell-free nucleosomes, MPO, neutrophil elastase, and cathepsin G are closely related to the severity of RA. Studies have shown that anti-TNF-α and anti-IL-6R treatments result in decreased formation of NETs and are accompanied by reduction of inflammation and serum markers, endothelial dysfunction, and suppression of immune cell activation (156, 157). The MPO-DNA complex in the serum of patients with RA is reported to be markedly higher than that in the healthy control group. Its levels are associated with the levels of anti-citrullinated protein antibodies (ACPA) and RF, and thus may prove be useful as a supplementary biomarker for clinical assessment of RA (158). The enhanced NETosis observed in the circulation and RA synovial fluid is related to ACPA and systemic inflammation markers. The serum and immunoglobulin components of RA patients with high levels of ACPA and RF evidently demonstrate enhanced NETosis. Furthermore, IL-8, IL-17A, and TNF-α have been shown to induce NETosis. In turn, NETosis containing citrullinated peptides is internalized and taken up by FLS through the RAGE-TLR9 pathway thereby upregulating the expression of MHC class II (MHCII). The MHCII molecule is then presented to CD4^+^ T cells, which strengthens the autoimmune B cell response and the production of ACPA. These events ultimately result in the induction of IL-6, IL-8, in addition to a variety of other chemokines and adhesion factors, to promote the formation of a vicious circle of inflammation (148, 156, 159, 160). Wright et al. have shown that low-density granulocytes (LDG) in RA exhibit lower TNFR1/2 mRNA and protein expression, indicating the presence of an immature neutrophil population in the peripheral blood, which may ultimately result in poor response to anti-TNF-α therapy (161). In addition, the level of calprotectin in RA has been shown to be elevated in the systemic and synovial fluid. However, their levels are markedly decreased with effective treatment. As a result, calprotectin can serve as a significant predictor for the progression of joint destruction and drug efficacy in RA (162). Anti-CCP, IgA, IgM, and RF-positive RA patients exhibit higher calprotectin levels as compared to the control groups that correlates with the baseline levels of inflammation markers, CRP, ESR, and anti-CCP (163). Consistently, a study by Bach et al. has demonstrated that the levels of calprotectin and NETs of neutrophils are markedly increased in RA patients, which represents transitional activation and cell death, respectively. The study further revealed that the calprotectin levels were significantly related to disease severity, and the levels of ACPA cooperatively enhanced the prediction of bone erosion and disability in patients with RA. In addition, NETosis is significantly associated with inflammatory disease markers and thus, can be used as a predictor of nodule development in patients with RA (164). Zhu et al. have shown that emodin, a natural anthraquinone derivative, can (1) reduce neutrophil infiltration and the release of pro-inflammatory factors, such as IL-6, IFN-γ, and TNF-α; (2) promote the expression of pro-apoptotic proteins, such as caspase-3 and Bax; and (3) inhibit the expression of anti-apoptotic protein Bcl-2 for promoting neutrophil apoptosis and inhibiting Atg5, LC3B, Beclin-1, and other autophagy protein levels by restraining autophagy and NETosis, which alleviates the symptoms RA in the mouse model of adjuvant-induced arthritis (AA) (165). Felty syndrome, a severe form of RA, is characterized by the presence of a large amount of anti-histone autoantibodies, which may be derived from neutrophil NETosis. Moreover, studies have shown that some circulating autoantibodies in Felty syndrome preferentially target PAD4-deaminated histones, such as H3, H4, H2A, and bind to activated neutrophils and NETs, further clarifying the role of NETosis in RA (166) (See **Figure 3**).

## Necroptosis in the Pathophysiology of Rheumatoid Arthritis

Necroptosis is essentially a PCD mediated by receptor-interacting protein kinase-1 (RIPK1), RIPK3, mixed lineage kinase-like (MLKL). The interaction between RIPK1 and RIPK3 is related to the crosstalk between necroptosis and apoptosis. Its morphological characteristics have been widely reviewed, and involve the release of cellular contents, ultimately mediating inflammation in RA (167–170). Neutrophils in the joints of RA patients activate RIPK1, RIPK3, and MLKL under the influence of CD44 and granulocyte-macrophage colony-stimulating factor (GM-CSF) to cause necroptosis, which can be blocked through the application of fibroblast activation protein-α (FAP-α) suggesting its importance as an potent drug for RA treatment (171). The expression of 14-3-3η can be detected in the synovium and serum of RA patients and is closely related to disease severity and the level of anti-cyclic citrullinated peptide antibodies. Macrophages in the RA synovium respond to TNF-α stimulation, and p-MLKL significantly increases the release of 14-3-3η in the induction of necroptosis (172). A spontaneous RA study involving a non-human primate (NHP) model showed that RIPK1 binds to voltage-dependent anion-selective channel 1 (VDAC1) in the heart causing its enhanced oligomerization. This ultimately increases the death and impairment of cardiomyocytes thereby resulting in aggravated RA-related heart function abnormalities (173). In addition, studies with articular cartilage in rats with AA and *in vitro* chondrocytes have shown an acid-sensitive ion channel (ASIC)-1a-mediated increase in the expression of RIPK1, RIPK3, and P-MLKL. Moreover, treatment with necrostatin (NST)-1s, a RIPK1 inhibitor, has been shown to reduce joint damage and inflammation in AA rats (174). Similarly, NST-1s can inhibit the expression of necroptosis-related molecules (RIPK1, RIPK3, and MLKL) in mice with experimental CIA arthritis, thereby reducing the population of osteoclasts, Th1, and Th17 cells; and augmenting the population of Th2 and Treg cells (175). Furthermore, the lack of interferon γ (IFN- γ) has also been reported to promote the expression of RIPK1, RIPK3 and MLKL in CIA mice, increase the number of Th17 cells, promote the release of IL17 and TNF-α, and activate STAT3, thereby aggravating inflammation and joint damage in RA (176).

**Figure 4:**
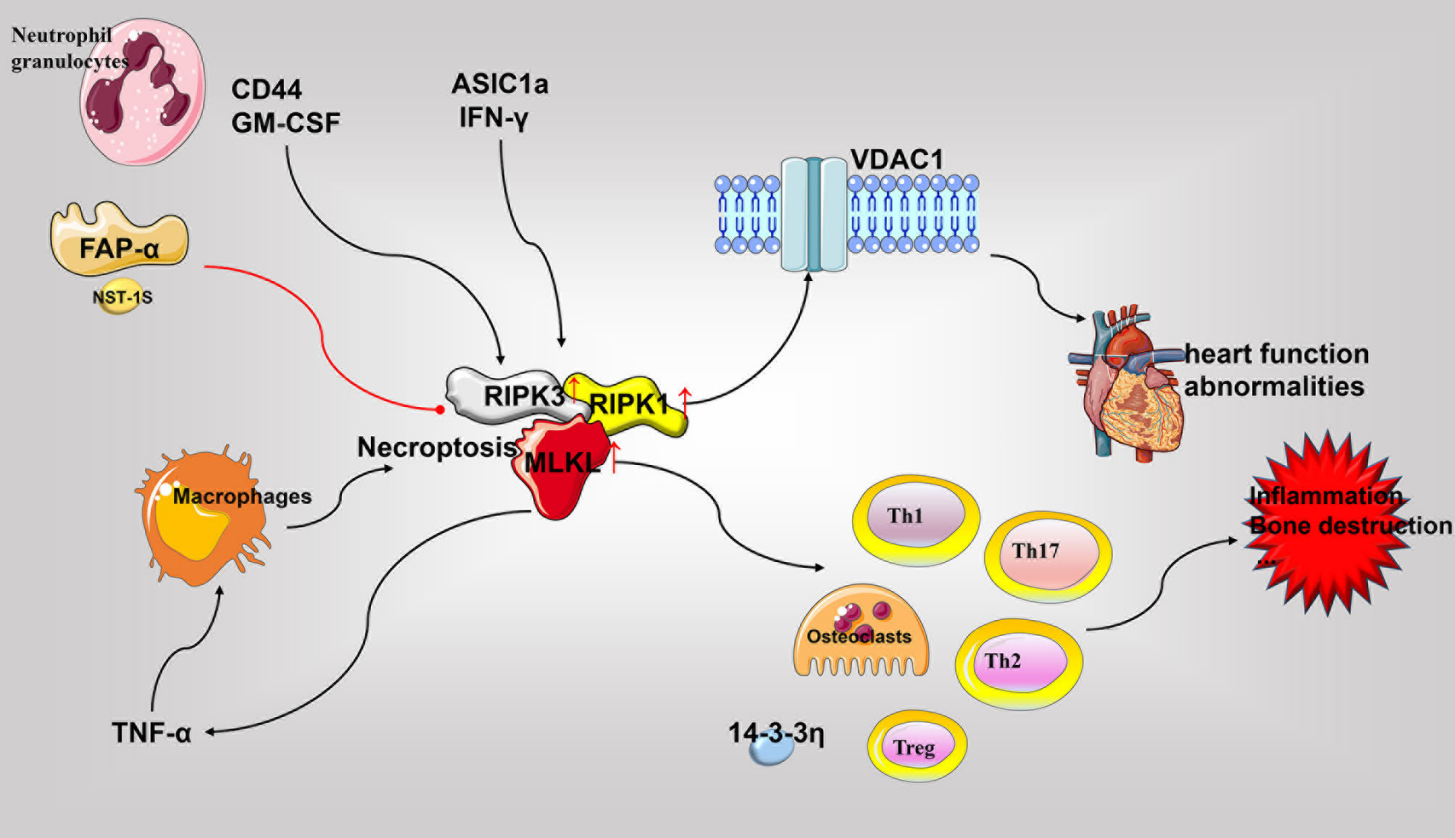
Role of necroptosis in the pathophysiology of rheumatoid arthritis. The critical molecules of necroptosis, RIPK1, RIPK3, and MLKL, are elevated in RA. Various cell types undergo necroptosis in response to diverse stimuli and release various pro-inflammatory mediators to promote inflammation, bone destruction, and other RA associated pathological processes. Necroptosis in cardiomyocytes may cause RA-related heart damage. Accordingly, inhibition of RIPK1 and other necroptotic molecules may be beneficial for disease treatment. GM-CSF, granulocyte-macrophage colony-stimulating factor; RIPK, receptor-interacting protein kinase; MLKL, pseudokinase mixed lineage kinase domain-like; TNF, tumor necrosis factor; VDAC1, voltage-dependent anion-selective channel 1.

Apoptosis and necroptosis are associated with each other in the progression of RA. Studies have shown that second mitochondria-derived activator of caspases (SMAC) mimetics (SMs) can inhibit the anti-apoptotic effect of Smac, which is a member of the IAP family protein, by competing with CIAP1/2 protein to induce the activation of caspase-3, caspase-7, caspase-8, caspase-9, and Fas; thereby facilitating the release of TNF-α; and *in vitro* activation of RIPK1, RIPK3, and MLKL in pro-inflammatory M1 macrophages to activate apoptosis and necroptosis. Blockade of the IAP and caspase pathways, facilitates M2c phenotype induction by SMs, resulting in necroptosis of M0 macrophages (177). Compared with etanercept, a TNF-α inhibitor, geldanamycin inhibits the RIPK1 and NF-KB activity while augmenting the activation of caspase-8 to promote MH7A cell apoptosis, and inhibit TNF-α-induced necroptosis. Thus, geldanamycin demonstrably results in better clinical outcomes and lowered synovial hyperplasia in CIA mouse model (178). A multicenter, randomized, double-blind, placebo-controlled clinical trial has evaluated the effect of the RIPK1 inhibitor GSK2982772 in RA. The study revealed that as compared with the placebo group, the inhibiter administered group demonstrated no significant differences in certain common clinical indicators and disease severity, such as DAS28-CRP and ACR20/50/70, (NCT02858492) (179) (See **Figure 4**).

## Pyroptosis in the Pathophysiology of Rheumatoid Arthritis

Pyroptosis is a type of pro-inflammatory PCD. Its morphological characteristics include membrane rupture and the release of cellular contents. Pyroptosis is mainly composed of ASC, caspase-1, and other proteins of the NLRP3 inflammasome, GSDMD, IL-1β, and IL-18. The characteristic purpose of inflammasome assembly is to resist pathogens that are harmful to the body and maintain homeostasis. However, when NLRP3 inflammasomes and pyroptosis are over-activated, the released inflammatory mediators can cause inflammation and damaging reactions. The role of inflammasomes in a variety of autoimmune inflammatory conditions has been widely reviewed. Here, we essentially discuss the connection of inflammasomes and pyroptosis with respect to prominent cell types known to play significant roles in the pathogenesis of RA including monocytes-macrophages, chondrocytes, CD4^+^ T cells, and FLS (180–183).

**Figure 5:**
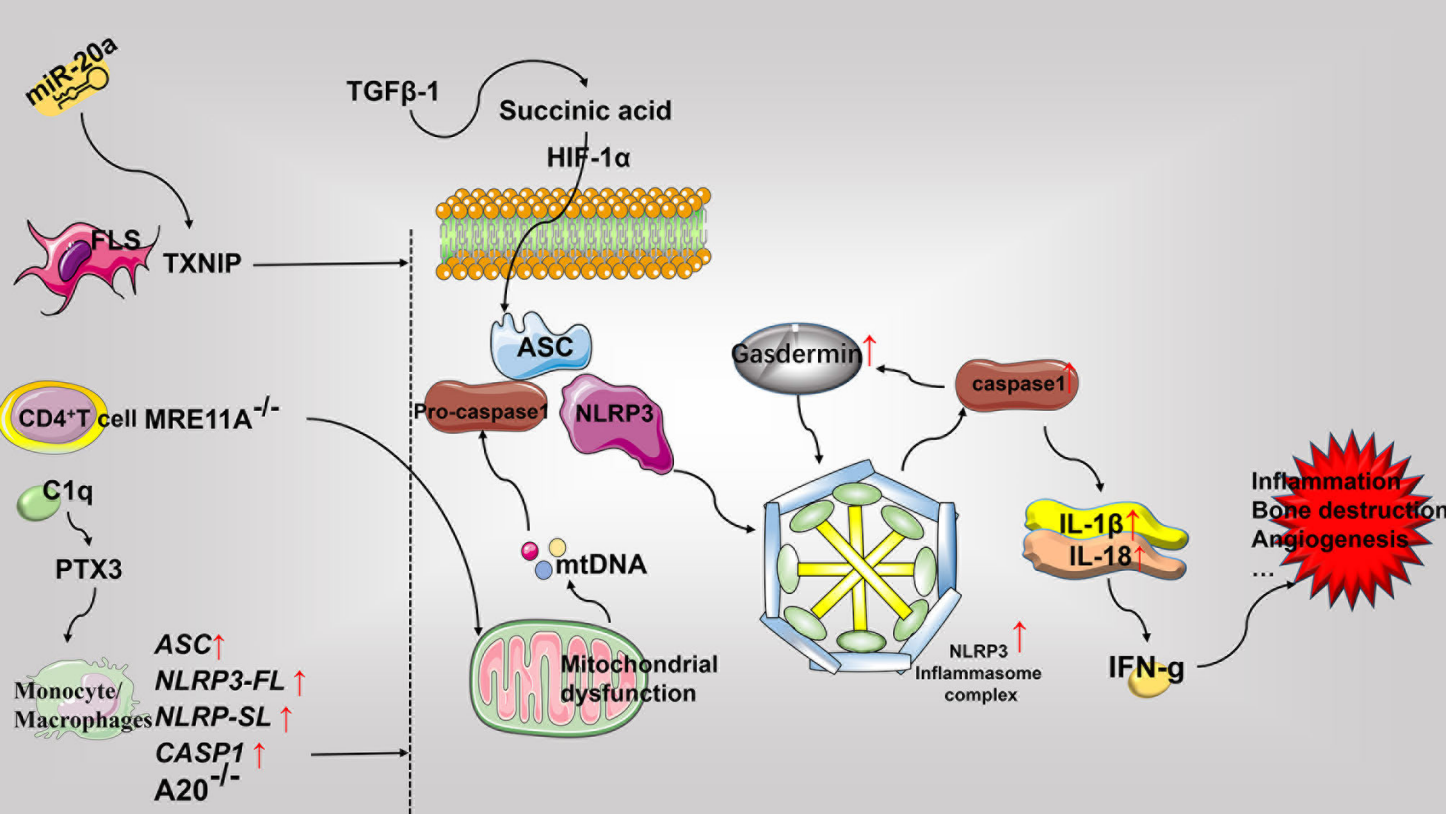
Role of pyroptosis in the pathophysiology of rheumatoid arthritis. Pyroptosis mainly involves the assembly of the NLRP3 inflammasome and the release of the pro-inflammatory mediators including IL-1β and IL-18. Most pyroptosis-related molecules are elevated in RA. Predominantly, the FLS, CD4^+^T cells, and monocytes-macrophages express different molecular patterns and promote pyroptosis, inflammation, bone destruction, and angiogenesis, that ultimately worsens exacerbates RA. FLS, fibroblast-like synoviocytes; PTX3, pentaxin 3; GSDMD, gasdermin D; mtDNA, mitochondrial DNA; TGF, transforming growth factor; HIF-1α, hypoxia-inducible factor-1α.

Elevated IL-1β and IL-18 levels are detected in the serum and synovial fluid of patients with RA (184). In RA synovium, transforming growth factor (TGF) β-1 reverses the activation of succinate dehydrogenase to increase succinate accumulation. This facilitates the assembly of NLRP3 inflammasomes *via* HIF-1α to activate pyroptosis for releasing IL-1β and IL-18 (185). Further, IL-18 reportedly induces inflammation by activating IFN-γ. Moreover, the expression of IL-18 is closely associated with the inflammation of synovial tissue in RA patients, and involved in the promotion of monocyte chemotaxis in addition to angiogenesis, thereby playing an important role in the induction of RA in experimental arthritis mouse models (183). Notably, TLR3 and TLR4 stimulation of patients with active RA has been documented to increase the levels of NLRP3, pyroptosis, and IL-1β released from whole blood cells (186), This is consistent with previous reports demonstrating that RA associated monocytes can release IL-1β through NLRP3-mediated pyroptosis (187). Furthermore, the expression of *ASC*, *NLRP3-FL*, *NLRP-SL*, and *CASP1* in the monocytes derived from RA patients as well as the serum levels of caspase-1 and IL-18 have been found to be elevated in RA patients as compared to the control group. Moreover, the SNPs of NLRP3 was also identified to be involved in determining the susceptibility to RA and resistance to TNF-α therapy (188). C1q in the serum of RA patients has been shown to promote the binding of pentaxin 3 (PTX3) to CD14 monocytes, resulting in the lysis of gasdermin D (GSDMD), activation of NLRP3 inflammasomes, swelling of cells into bullae, and ultimate release of caspase-1, TNF-α, IL -6, and IL-1β concurrently to promote pyroptosis (189). In addition, studies have found that M1 macrophages and pyroptosis in the CIA model are the main sources of inflammatory factors. Punicalagin (PUN) is an active substance extracted from pomegranate peel. Its treatment has been documented to transform macrophages into anti-inflammatory phenotypes to reduce the release of inducible nitric oxide synthase (iNOS), increase the levels of IL-10 and other anti-inflammatory mediators for inhibiting pyroptosis, and facilitate the NLRP3 -mediated release of IL-1β and IL-18 in addition to caspase-1, ultimately resulting in the inhibition of inflammation (190). The A20/TNFAIP3 mouse model has been reported to develop spontaneous erosive polyarthritis, in which A20-deficient macrophages exhibit enhanced NLRP3, caspase-1, IL-1β, and pyroptosis. Furthermore, the absence of NLRP3, caspase-1, and IL-1R notably inhibits the inflammation and joint destruction in A20/TNFAIP3 mice (191). Similarly, *IL-1β* gene-deficient mice are completely protected from chronic inflammation-mediated joint destruction and damage (192). The frequency of caspase-1 activation by CD4^+^ T cells in patients with RA is higher than that in healthy controls. Defects in MRE11A, the DNA repair nuclease, has been reported to cause CD4^+^ T cell mitochondrial dysfunction and the leakage of mitochondrial DNA (mtDNA), leading to the assembly of NLRP3 inflammasomes, activation of caspase-1, and pyroptosis. Moreover, the overexpression of MRE11A reverses these effects associated with defective MRE11A. Thus, MRE11A participates in RA associated inflammation by mediating mitochondrial homeostasis and pyroptosis (193). ASIC1a not only participates in the regulation of necroptosis in RA but also mediates pyroptosis in AA model of chondrocytes by promoting the assembly of Ca^2+^-related NLRP3 inflammasomes, the expression of caspase-1, and the release of IL-1β and IL-18 (194). The primary rat articular chondrocytes acidified *in vitro* demonstrate increased ASIC1a, calpain-2, and calcineurin, NLRP3, caspase-1 expression, and pyroptosis. In contrast, the inhibition of ASIC1a, calpain-2, and calcineurin reduces pyroptosis, IL-1β, and other inflammatory mediators (195). Furthermore, the overexpression of miR-20a has been documented to inhibit FLS pyroptosis in the AA model by downregulating the expression of TXNIP, resulting in reduced levels of NLRP3, ASC, caspase-1, and IL-1β (196) (See **Figure 5**).

## Conclusion

In a healthy organism, cell death is important for the proper maintenance of the balance between the body and tissue cells. When any part of the pathway becomes defective, the balance between survival and death of various cells is disrupted, which results in manifestations of abnormal patho-physiological conditions. Several studies have shown that the imbalance of PCD in promoting the chronic inflammation of the synovial membrane, joint erosion, and destruction, as well as angiogenesis plays an important role in promoting RA. In this review, we predominantly discuss the relationship between a variety of PCD and RA pathogenesis. We have first discussed upon how the insufficient apoptosis of FLS leads to abnormal proliferation of synovium, autoimmune T cells, B cells, inflammatory monocytes, and inflammation. Further, we have deliberated over the irregular apoptotic pattern of autoimmune T cells, B cells, inflammatory monocytes, inflammatory macrophages, and osteoclasts, which results in prolonged cell survival, thereby facilitating continuous presentation of autoantigens, the release of inflammatory factors, and the destruction of bones and joints. The excessive apoptosis of osteoblasts leads to a decrease in bone formation. Secondly, we reviewed the role of autophagy in the progression of RA in the context of various cell types involved in the process. The autophagy of FLS antagonizes apoptosis and contributes to the abnormal proliferation of synovium. Furthermore, the autophagy of a variety of APC cells enhances the presentation of autoantigens and the activation of CD4^+^ T cells. Moreover, the abnormal autophagy patterns of osteoblasts and osteoclasts are attributable to the low bone mass and increased bone resorption in RA, respectively. Additionally, the autophagy of other cells has different degrees of contribution to the pathological process of RA. Finally, we have described the role of the three forms of PCD, namely, NETosis, Necroptosis, and Pyroptosis in the pathogenesis of RA. NETosis of neutrophils is mainly involved in the formation of citrullinated proteins in RA. Necroptosis of a variety of cells primarily leads to the release of cell contents, which further promotes chronic inflammation. In addition, pyroptosis of various cells types mainly releases pro-inflammatory cytokines (IL-18 and IL-1β) and leading to the formation of inflammasomes that mediate RA associated chronic inflammation. Exhaustive literature mining reveals that multiple PCDs involved in the pathogenesis of RA communicate with each other and cooperatively promote the multiple pathological processes associated with RA. Rapid progress in the field of cell death research in the last decade, has led to the development of various experimental methods and technologies that can be helpful in detecting the link between PCD-related molecules and RA. The various studies in the field have achieved certain degree of progress, which has resulted in the identification of some biomarkers associated with the pathogenesis of RA (**Table 1**).In conclusion, in this review, we have summarized a variety of abnormal PCD and their mechanisms in RA in addition to provided corresponding clinical strategies for their application (**Table 2**). With the development and application of various PCD inhibitors and agonists, researchers can conduct an in-depth examination of the role of various PCDs in RA progression. Thus, future research directions will be based on the efficacy of preclinical experiments, which should essentially involve comprehensive experimental programs devoted to the application of multiple agonists and inhibitors for validating the effects of various PCD-related molecules on different cell types associated with RA. The immense potential attributed to this line of research is based on the complexity of RA and PCD mechanisms which can facilitate the expansion of existing repertoire of promising drug candidates for the development of effective RA drugs. The ultimate purpose of designing such treatment strategies is to rebalance the abnormal cell survival and death to combat the consequences of excessive or insufficient PCD in the various cell types involved in RA progression.

**Table 1:** Rheumatoid arthritis related biomarkers of programmed cell death.

Name	Potential role	Ref.
MCL-1	It is associated with the degree of inflammation and TNF-α level in addition to inhibiting the apoptosis of FLS.	(21)
PDCD5	It is negatively correlated with CRP, ESR, IL-17, and TNF-α levels.	(49, 50)
β2-R	It is negatively correlated with disease severity.	(89)
Cell-free nucleosomes	It is associated with disease severity.	(156, 157)
Myeloperoxidase	It is associated with disease severity.	(156, 157)
Neutrophil elastase	It is associated with disease severity.	(156, 157)
MPO-DNA complex	It is associated with ACPA and RF levels.	(158)
Calprotectin	It is associated with the serum markers of inflammation, CRP, ESR, and baseline levels of anti-CCP, as well as predicting the progression of joint destruction and drug efficacy.	(162, 163)
14-3-3η	It is closely associated with disease severity and anti-cyclic citrullinated peptide antibody levels.	(172)
IL-18	It is associated with inflammation.	(183)
SNP of NLRP3	It is related to RA susceptibility and response to anti-TNF-α therapy.	(188)

**Table 2:** Rheumatoid arthritis related clinical strategies for targeting programmed cell death.

Items	Clinical strategies
Apoptosis	Increasing apoptotic sensitivity of FLS, autoimmune T cells, B cells, pro-inflammatory monocytes-macrophages, and osteoclasts; while inhibiting excessive apoptosis of osteoblasts/chondrocytes.
NETosis	Inhibiting the formation of excessive NETosis, reducing the release of pro-inflammatory biological mediators, and converting the death form of neutrophils into a safer form of cell death
Necroptosis	By inhibiting the critical molecules of necroptosis such as RIPK1, RIPK3, and MLKL; reduce the release of pro-inflammatory mediators in various cells, and it may be more advantageous to transform into RIPK1-mediated apoptosis.
Pyroptosis	By inhibiting the formation of the NLRP3 inflammasome directly inhibits pyroptosis and reduces the release of pro-inflammatory IL-1β and IL-18, thereby reducing inflammation and joint damage.
Autophagy	Targeting therapy on autophagy still needs precautions. Apparently, inhibiting autophagy and increasing apoptosis is more advantageous, and hence further experimental confirmation is required.

## Author Contributions

JZ is responsible for the collection of data, collation, and writing of the original manuscript. PJ is responsible for the organization of the original manuscript. SG, SS, and DH are responsible for the concept development, revision, and review of the manuscript. All authors contributed to the article and approved the submitted version.

## Funding

This work was funded by the National Natural Science Funds of China (81774114), Shanghai Chinese Medicine Development Office, Shanghai Chinese and Western Medicine Clinical Pilot Project (ZY(2018-2020)-FWTX-1010), Shanghai Chinese Medicine Development Office, Shanghai Traditional Chinese Medicine Specialty Alliance Project (ZY(2018-2020)-FWTX-4017), National Administration of Traditional Chinese Medicine, and Regional Chinese Medicine (Specialist) Diagnosis and Treatment Center Construction Project-Rheumatology.

## Conflict of Interest

The authors declare that the research was conducted in the absence of any commercial or financial relationships that could be construed as a potential conflict of interest.

## Publisher’s Note

All claims expressed in this article are solely those of the authors and do not necessarily represent those of their affiliated organizations, or those of the publisher, the editors and the reviewers. Any product that may be evaluated in this article, or claim that may be made by its manufacturer, is not guaranteed or endorsed by the publisher.

## Abbreviations

RA, rheumatoid arthritis; PCD, programmed cell death; AICD, activation-induced cell death; FLS, fibroblasts like synoviocytes; Bcl-2, B cell lymphoma/leukemia-2; Bax, accumulation of Bcl-2–associated X protein; Bak, Bcl-2 homologous antagonist/killer; Cyt c, cytochrome c; TNF, tumor necrosis factor; TNFR1, tumor necrosis factor receptor 1; STAT3, activators of transcription 3; HSP70, heat-shock-protein-70; PBL, peripheral blood lymphocyte; PADI4, peptidylarginine deiminase IV; NSAIDs, non-steroidal anti-inflammatory drugs; PPAR gamma, peroxisome proliferator-activated receptor gamma; IκB, the intracellular calcium, phosphorylated I-kappa-B; IKK, the intracellular calcium, phosphorylated I-kappa-B (IκB) kinase; NF-κB, nuclear factor kappa-light-chain enhancer of activated B cells; AA, adjuvant arthritis; TIPE2, TNF-alpha-induced protein-8-like-2; DR5, Tumor necrosis factor (TNF)-related apoptosis-inducing ligand (TRAIL) receptor 2; MCL-1, myelogenous cell leukemia-1; Bim, Bcl-2 interacting mediator of cell death; SYVN1, the E3 ubiquitin ligase synoviolin; IRE1, inositol-requiring enzyme 1; CIA, collagen-induced arthritis; sFas, soluble Fas; mFas, membrane-bound Fas; ERK, extracellular signal-regulated kinase; PI3K, phosphatidylinositol 3-kinase; MMP, matrix metalloproteinase; IPT, imperatorin; TIMP-3, tissue inhibitor of metalloproteinases-3; CIP2A, cancerous inhibitor of protein phosphatase 2A; PDGF, platelet-derived growth factor; HIF-1α, hypoxia-inducible factor-1α; BAFF, B-cell activating factor; ASIC3, acid-sensing ion channel 3; TRPV1, transient receptor potential vanilloid 1; ROS, reactive oxygen species; NO, nitric oxide; TRPM8, transient receptor potential melastatin subtype 8; POGel, prolonged O2/Ca^2+^-supporting phototherapy hydrogel; CHOP, EBP-homologous protein; GA, Geldanamycin; PDCD5, programmed cell death 5; ALLN, calpain inhibitor 1; CBP, CREB binding protein; IAPs, inhibitors of apoptosis proteins; DC, dendritic cells; IL, Interleukin; RANKL, receptor activator of nuclear factor (NF)-κB ligand; TRAIL-R2, TNF-related apoptosis-inducing ligand-receptor 2; NADPH, nicotinamide adenine dinucleotide phosphate; PARP-1, poly (ADP-ribose) polymerase-1; CARD6, caspase recruitment domain protein 6; TRAF2, tumor necrosis factor receptor -associated factor-2; HOCl, hypochlorous acid; MPCs, mesenchymal progenitor cells; NETs, neutrophil extracellular traps; MPO, myeloperoxidase; NE, neutrophil elastase; CarP, carbamylated protein; chcap-18, cathelicidin human cationic antimicrobial protein-18; PAD, peptidylarginine deiminase; MHCII, MHC class II; LDG, low-density granulocytes; RF, rheumatoid factor; anti-CCP, anti-cyclic citrullinated peptide; ACPA, anti-citrullinated protein antibodies; AA, adjuvant-induced arthritis; RIPK, receptor-interacting protein kinase; MLKL, pseudokinase mixed lineage kinase domain-like; FAP-α, fibroblast activation protein-α; NHP, non-human primate; VDAC1, voltage-dependent anion-selective channel 1; ASICs, acid-sensitive ion channels; IFN-γ, interferon γ; SMAC, second mitochondria-derived activator of caspases; SMs, SMAC mimetics; PTX3, pentaxin 3; GSDMD, gasdermin D; PUN, punicalagin; mtDNA, mitochondrial DNA; PTPN2, phosphatase nonreceptor type 2; OA, osteoarthritis; PFKFB3, 6-phosphofructo-2-kinase/fructose-2,6-bisphosphatase 3; SERCA, sarcoplasmic/endoplasmic reticulum Ca ATPase pump; AIA, adjuvant-induced arthritis; LC3b, light chain 3b; ULK-1, UNC51-like kinase 1; TLR, Toll-like receptor; GM-CSF, granulocyte-macrophage colony-stimulating factor; TGF, transforming growth factor; BID, the BH3 interacting-domain death agonist.

## Glossary

**Table:**

RA	rheumatoid arthritis
PCD	programmed cell death
AICD	activation-induced cell death
FLS	fibroblasts like synoviocytes
Bcl-2	B cell lymphoma/leukemia-2
Bax	accumulation of Bcl-2–associated X protein
Bak	Bcl-2 homologous antagonist/killer
Cyt c	cytochrome c
TNF	tumor necrosis factor
TNFR1	tumor necrosis factor receptor 1
STAT3	activators of transcription 3
HSP70	heat-shock-protein-70
PBL	peripheral blood lymphocyte
PADI4	peptidylarginine deiminase IV
NSAIDs	non-steroidal anti-inflammatory drugs
PPAR gamma	peroxisome proliferator-activated receptor gamma
IκB	the intracellular calcium, phosphorylated I-kappa-B
IKK	the intracellular calcium, phosphorylated I-kappa-B (IκB) kinase
NF-κB	nuclear factor kappa-light-chain enhancer of activated B cells
AA	adjuvant arthritis
TIPE2	TNF-alpha-induced protein-8-like-2
DR5	Tumor necrosis factor (TNF)-related apoptosis-inducing ligand (TRAIL) receptor 2
MCL-1	myelogenous cell leukemia-1
Bim	Bcl-2 interacting mediator of cell death
SYVN1	the E3 ubiquitin ligase synoviolin
IRE1	inositol-requiring enzyme 1
CIA	collagen-induced arthritis
sFas	soluble Fas
mFas	membrane-bound Fas
ERK	extracellular signal-regulated kinase
PI3K	phosphatidylinositol 3-kinase
MMP	matrix metalloproteinase
IPT	imperatorin
TIMP-3	tissue inhibitor of metalloproteinases-3
CIP2A	cancerous inhibitor of protein phosphatase 2A
PDGF	platelet-derived growth factor
HIF-1α	hypoxia-inducible factor-1α
BAFF	B-cell activating factor
ASIC3	acid-sensing ion channel 3
TRPV1	transient receptor potential vanilloid 1
ROS	reactive oxygen species
NO	nitric oxide
TRPM8	transient receptor potential melastatin subtype 8
POGel	prolonged O2/Ca^2+^-supporting phototherapy hydrogel
CHOP	EBP-homologous protein
GA	Geldanamycin
PDCD5	programmed cell death 5
ALLN	calpain inhibitor 1
CBP	CREB binding protein
IAPs	inhibitors of apoptosis proteins
DC	dendritic cells
IL	Interleukin
RANKL	receptor activator of nuclear factor (NF)-κB ligand
TRAIL-R2	TNF-related apoptosis-inducing ligand-receptor 2
NADPH	nicotinamide adenine dinucleotide phosphate
PARP-1	poly (ADP-ribose) polymerase-1
CARD6	caspase recruitment domain protein 6
TRAF2	tumor necrosis factor receptor -associated factor-2
HOCl	hypochlorous acid
MPCs	mesenchymal progenitor cells
NETs	neutrophil extracellular traps
MPO	myeloperoxidase
NE	neutrophil elastase
CarP	carbamylated protein
chcap-18	cathelicidin human cationic antimicrobial protein-18
PAD	peptidylarginine deiminase
MHCII	MHC class II
LDG	low-density granulocytes
RF	rheumatoid factor
anti-CCP	anti-cyclic citrullinated peptide
ACPA	anti-citrullinated protein antibodies
AA	adjuvant-induced arthritis
RIPK	receptor-interacting protein kinase
MLKL	pseudokinase mixed lineage kinase domain-like
FAP-α	fibroblast activation protein-α
NHP	non-human primate
VDAC1	voltage-dependent anion-selective channel 1
ASICs	acid-sensitive ion channels
IFN-γ	interferon γ
SMAC	second mitochondria-derived activator of caspases
SMs	SMAC mimetics
PTX3	pentaxin 3
GSDMD	gasdermin D
PUN	punicalagin
mtDNA	mitochondrial DNA
PTPN2	phosphatase nonreceptor type 2
OA	osteoarthritis
PFKFB3	6-phosphofructo-2-kinase/fructose-2,6-bisphosphatase 3
SERCA	sarcoplasmic/endoplasmic reticulum Ca ATPase pump
AIA	adjuvant-induced arthritis
LC3b	light chain 3b
ULK-1	UNC51-like kinase 1
TLR	Toll-like receptor
GM-CSF	granulocyte-macrophage colony-stimulating factor
TGF	transforming growth factor
BID	the BH3 interacting-domain death agonist
